# Structural modeling of Chinese students’ academic achievement identity and basic psychological needs: do academic self-efficacy, and mindfulness play a mediating role?

**DOI:** 10.1186/s40359-024-01571-6

**Published:** 2024-03-13

**Authors:** Shuya Chen

**Affiliations:** Basic Courses Teaching Department, Henan Judicial Police Vocational College, 450016 Zhengzhou, Henan, China

**Keywords:** Academic achievement identity, Basic psychological needs, Mindfulness, Academic self-efficacy

## Abstract

**Background:**

Mindfulness and academic self-efficacy were proposed as mediating variables, with successful academic identity as an exogenous variable. The backdrop for this research centers on the significance of psychological factors in shaping academic identity among college students.

**Objectives:**

The primary aim of the research was to investigate the relationship between fundamental psychological needs, mindfulness, academic self-efficacy, and successful academic identity. Specifically, the study explored the direct and indirect impacts of basic psychological needs on successful academic identity mediated by mindfulness and academic self-efficacy.

**Method:**

The research employed a descriptive method, utilizing correlational studies and structural equation modeling. A sample of 355 college students at Henan Judicial Police Vocational College, Henan, China, was randomly selected through multistage cluster sampling. Data were collected using established scales, including the Basic Psychological Needs Scale, Baer Mindfulness Scale, Jenkins and Morgan Academic Self-Efficacy Scale, and Vas and Isakson Successful Academic Identity Scale. The data analysis was conducted using AMOS 22 software.

**Findings:**

The research findings revealed that fundamental psychological needs directly and indirectly significantly impact successful academic identity. Mindfulness played a mediating role in this relationship. However, academic self-efficacy did not considerably mediate the influence of fundamental psychological needs on successful academic identity (*p* > 0.05). These results highlight the complex dynamics between psychological needs, mindfulness, academic self-efficacy, and successful academic identity among college students in the specified academic year.

**Conclusions:**

The findings suggest targeted interventions, such as workshops for families and teachers to address basic needs and psychologist and school counselor interventions to increase mindfulness. Additionally, organizing educational classes is imperative for fostering a supportive environment conducive to successful academic identity among college students.

## Introduction

Adolescence is one of the most stressful developmental stages, leading to extensive physiological and psychological changes. This issue is the most significant challenge during adolescence [1]. in identity formation. Identity is a central concept in Erikson’s psychosocial theory and is defined as a relatively stable emotional sense of self [2]. Presenting a complete and comprehensive definition of identity is almost impossible, and arriving at a singular meaning is impossible. This is because identity has various dimensions that emerge from different contexts [3]. One of the contexts where adolescents spend a significant portion of their daily lives is the school environment, with the identity associated being recognized as academic identity [4]. As individuals transition into adolescence, they ask themselves different questions within the school environment. Questions like: “What to study?”, “How to study?”, “Where to study?”, “Why study?” and so on. These questions arise from cognitive changes during this stage and the pursuit of independence and unified identity, often accompanied by stress and anxiety. By assessing their abilities, beliefs, and past experiences and considering others’ perceptions of themselves, learners establish an organized structure through which they can clearly define themselves [5]. If based on exploration and commitment, this clear and coherent structure of self is termed a successful academic identity [4]. Therefore, students’ level of competence, autonomy, goals, self-efficacy beliefs, and prevalent emotions in the classroom reflect their academic identity [6]. Was and Isakson [4], inspired by Marcia’s identity statuses [7] and based on the two factors of exploration and commitment, defined successful academic identity as follows: It is the most adaptive style where the learner successfully navigates the process of seeking school-related values and, by determining values and goals, commits to them earnestly.

Glasser [8] explained in his identity theory that the characteristics of individuals with a successful identity include having an emotional connection with themselves, establishing interaction with others, feeling love and appreciation, and seeing themselves as a “coherent whole”. behave [9]..In addition, Deci and Ryan [10] introduce dignity, independence and the three basic needs of autonomy, relatedness and competence as essential components. It refers to the need for freedom, the sense of choice and autonomy in initiating, continuing, terminating and regulating activities [11]. It also refers to the need for competence, which involves utilizing existing abilities and perceiving these capabilities to influence the surrounding environment [12]. The need for competence becomes doubly crucial because dissatisfaction leads to a sense of weakness in agency and poses a threat to self-efficacy and the sense of capability in performing and organizing actions [11]. The need for connection, belonging to a group, being noticed, respected, and valued by others is highlighted [13].

Fundamental psychological needs, among the most crucial human needs, significantly impact individual mental health and functioning, existing in all individuals where their satisfaction is essential [14]. According to self-determination theory, individuals pursue goals that satisfy their psychological needs. When these psychological needs (autonomy, relatedness, and competence) are met, they engage in inner motivation and exploration [15]. As observed, Glasser [8], cited in Shafiee Abadi & Nasiri [9], lists characteristics for successful identity that are nearly synonymous with those outlined by Deci and Ryan [10] as part of fundamental psychological needs. Accordingly, fundamental psychological needs have an impact on successful academic identity. In this vein, Faye and Sharpe’s research [16] examines the positive effectiveness of autonomy and competence on identity. Similarly, the effectiveness of fundamental psychological needs on informational and normative identity styles was explored by Ghalamali Lavasani, et al. [17]. Naser Zaeim’s research [18] reported the positive indirect effectiveness of autonomy and competence on academic identity, and Moghaddas and Tabaei utilitarian research [19] the positive correlation between the need for competence and the need for connection with the internalization of ethical identity. In addition to fundamental psychological needs, mindfulness may mediate successful academic identity. Fulfilling fundamental psychological needs and mindfulness increases resilience and adaptability in stressful situations, making managing such situations more efficacious [20].

Numerous studies have also shown that mindfulness exercises increase self-confidence and physical and mental well-being. However, an important aspect overlooked in these studies is how mindfulness exerts its influence. To answer this question, a comprehensive theory must be formulated that helps explain this aspect. So far, no theories have been presented about this. Kabat-Zinn [21] believes that the effectiveness of mindfulness relies on physical relaxation and profound cognitive and behavioral changes. Mindfulness is a form of awareness in which a person pays targeted attention to events and experiences. Mindfulness emphasizes the present moment and the individual does not dwell on the past or the future, but rather accepts whatever is happening (the experience) without judgment [22]. If we combine all definitions of mindfulness, we would come up with three characteristics in almost all of them: (a) It is a state of alertness, (b) tt focuses on the present moment, and (c) it focuses attention on internal and external stimuli [23].

When individuals are self-aware in the moment and in control of their emotions, feelings, thoughts, and behaviors without judgment or bias, they demonstrate high levels of mindfulness [24]. Through increased attention and awareness of emotions, mindfulness improves a person’s cognitive understanding of their abilities and proves to be useful and constructive in various areas, including academic activities [25]. Individuals who exhibit such qualities also act mindfully in academic environments when tackling academic tasks without fixation. With increased mindfulness and thoughtful performance, cognitive abilities increase and the performance of the cognitive system improves, preventing cognitive overload and redundancy when tackling upcoming problem-solving tasks [26]. Students who meet their basic psychological needs design and guide their goals; Mindfulness in this process increases momentary attention and leads to review and adjustment of students’ goals and behaviors to better achieve their goals [26].

The satisfaction of fundamental psychological needs emerges as a significant factor closely linked to engagement in learning, as indicated by studies by Wilson et al. [27] and Yu et al. [28]. According to the Self-Determination Theory (SDT), postulated by Ryan and Deci [29], competence, autonomy, and relatedness constitute the three basic psychological needs influencing various individuals’ adaptive behaviors and psychological outcomes. Niemiec and Ryan [30] found a positive association between students’ perception of satisfaction of these basic psychological needs and their levels of academic well-being and school engagement. In specific learning environments, when students perceive themselves as competent, autonomous, and connected to their surroundings, their intrinsic motivation is activated, driving them to actively participate in academic tasks and attain higher performance, as noted by Sansone and Harackiewicz [31].

Another critical facilitator of learning is academic self-efficacy. Previous studies, such as those by Caraway et al. [32] and Bassi et al. [33], have suggested that students with high levels of academic self-efficacy exhibit increased engagement in learning, while those with lower levels tend to display indifference in class. This discrepancy is attributed to the motivational function of academic self-efficacy, defined as one’s confidence in their ability to accomplish academic tasks [34]. Students with a heightened sense of academic self-efficacy are motivated to employ more learning strategies, enhance cognitive competence [35], and demonstrate increased effort and persistence when faced with learning challenges [36].

By examining the definition and characteristics of self-efficacious individuals, meeting fundamental psychological needs such as competence enhances students’ efficacy in various domains, notably in academics. Perceiving this self-efficacy makes learners confident in their abilities, facing academic challenges with trust and without anxiety, thus progressing towards a successful academic identity.

Such a student, endowed with high self-efficacy, resiliently withstands external negative feedback by fulfilling the need for independence, organizing, and managing their cognitive system [37], ultimately achieving the outcome termed as a successful academic identity in this process. On the one hand, the significance of adolescence in shaping values and goals, coupled with the prevalent pressures and crises in this developmental stage [1], as well as the oversight of the student community in most studies on academic identity [38], designates college students as the optimal demographic for such research [38]. The absence of substantial research in modeling successful academic identity, both domestically and internationally, and the significance of societal influence on learner perception and its role in academic identity are among the critical factors that necessitate the importance of such research.

**Fig. 1:**
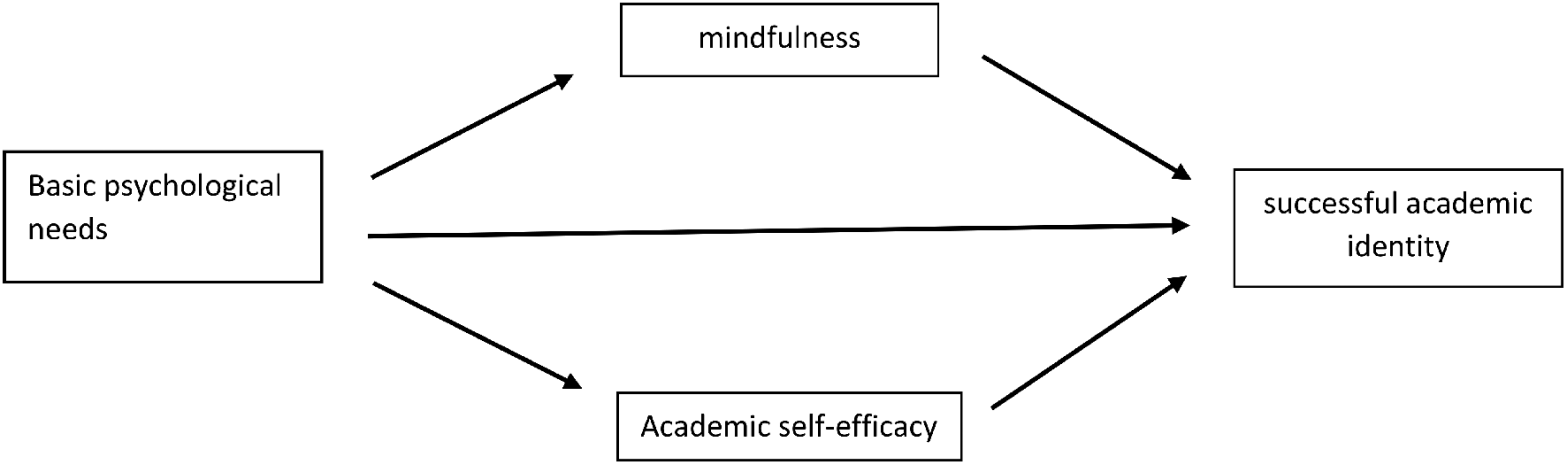
Proposed research model

Therefore, conducting a study titled ‘Structural Model of Successful Academic Identity Based on Fundamental Psychological Needs, Mediated by Mindfulness and Academic Self-Efficacy’ becomes imperative [39]. This study aims to determine whether fundamental psychological needs as predictive variables, mindfulness, and academic self-efficacy as mediating variables impact successful academic identity. The proposed research model is shown in Fig. 1.

## Research hypotheses

Based on the existing gap and the related literature review, the following hypotheses are stated:

### H1

Basic psychological needs have significant effect on the students’ mindfulness.

### H2

Basic psychological needs have significant effect on the students’ academic self-efficacy.

### H3

Basic psychological needs have significant effect on the students’ successful academic identity.

### H4

Basic psychological needs have significant effect on the students’ successful academic identity through the mediating variable of mindfulness.

### H5

Basic psychological needs have significant effect on the students’ successful academic identity through the mediating variable of academic self-efficacy.

### H6

Students’ mindfulness has significant effect on their successful academic identity.

### H7

Students’ academic self-efficacy has significant effect on their successful academic identity.

## Method

The present study used a descriptive method and utilized correlational research. All college students of Henan Judicial Police Vocational College studying in the 2022–2023 academic year constitute the research population (taking into account potential dropouts due to incomplete or distorted questionnaires). The sample size was determined based on a minimum of 15 cases per indicator, with an emphasis on large samples in structural equation modeling studies [40]. Taking into account the possibility of sample reduction due to incomplete or distorted graduation requirements, a total of 400 students were selected using multi-stage cluster random sampling. First, the Judicial Police Vocational School was randomly selected from four colleges in Henan. Two classes from each school were then randomly selected. Subsequently, 60 incomplete or distorted questionnaires were excluded from the research process. The collected data were analyzed by structural equation modeling using AMOS and SPSS software.

### Instruments

The following tools were used to gather the data.

#### Basic psychological needs

This tool was developed by La Guardia et al. [41], comprising 21 items categorized into three subscales: autonomy (items 1, 4, 8, 11, 17, and 20, with an example item being ‘I prefer to make decisions in my life on my own’), relatedness (items 2, 6, 7, 9, 12, 14, 16, 18, and 21, with an example item being ‘I care about the people I interact with’), and competence (items 3, 5, 10, 13, 15, and 19, with an example item being ‘I often don’t feel capable and efficient’). These items collectively form the respective subscales. The scoring of this tool is on a seven-point Likert scale (Not accurate at all: 1 to 7, very true:). Items 3, 4, 7, 11, 15, 16, 18, 19, and 20 are reverse-scored. In this tool, the minimum achievable score is 21, and the maximum is 147. Scores between 21 and 42 indicate low scores, 42 to 105 indicate moderate scores, and scores higher than 105 indicate high scores. La Guardia et al. [41] obtained satisfactory validity and an internal consistency reliability of 0.91. Within the country, Besharat and Ranjbar Kalagheri [42] calculated the reliability of this tool using the test-retest method to be between 0.67 and 0.77. Gholamali Lavasani et al., [43] reported the reliability using Cronbach’s alpha coefficient to range from 0.78 to 0.89. In the current study, Cronbach’s alpha coefficient was used to calculate reliability, resulting in a coefficient of 0.70.

#### Mindfulness scale

This tool was developed by Baer and colleagues in 2000. It comprises 39 items divided into five observation dimensions (items 1, 6, 8, 11, 15, 20, 26, 31 and 36 with an example item being ‘When I am walking, I deliberately notice the sensations of my body moving’, are examples of items in this subscale), concurrent action with vigilance which consists of items( 5, 8, 13, 18, 23, 28, 34, and 38, with an example item being ‘When I’m doing tasks, my mind wanders and I lose track easily.‘), non-judgmental experience of inner sensations which comprises (items 3, 10, 14, 17, 25, 30, 35, and 39, with an example item being ‘I criticize myself for having inappropriate or illogical emotions,’ are examples of items in this subscale.),description which includes (items 2, 7, 12, 22, 27, 32, and 37, with an example item being ‘I’m good at finding words to express my feelings.’ are examples of items in this subscale.), non-reactivity which encompasses (items 4, 9, 19, 21, 24, 29, and 33, with an example item being ‘I understand my emotions and feelings without the need to react to them.’ are examples of items in this subscale.) which was constructed and scored on a five-point Likert scale ranging from ‘very rarely: 1’ to ‘often: 5’. In this instrument, the minimum score achievable is 39, and the maximum score is 195. A higher score indicates higher levels of mindfulness. Baer et al. [44] reported satisfactory validity and reliability for this instrument, ranging from 0.75 to 0.91 using Cronbach’s alpha. Ahmadwand et al. [45] reported the reliability within the country using Cronbach’s alpha ranging from 0.55 to 0.83. In the present study, Cronbach’s alpha coefficient was used to calculate reliability, resulting in a coefficient of 0.74.

### The academic self-efficacy scale

This tool was developed by Jinks and Morgan [46], comprising 30 items categorized into three subscales: Structure, including Items 3, 4, 8, 12, 13, 15, 16, 17, 20, 23, 24, 28, and 29 compose the ‘Unimportance’ subscale, with an example item being ‘It does not matter to anybody even if I do well in school. The ‘Talent’ subscale includes items 6, 7, 10, 11, 14, 18, 19, 21, 22, 25, 26, 27, and 30, with an example item being ‘I am good at literary reading.’ The ‘Effort’ subscale comprises items 1, 2, 5, and 9, with an example item being ‘I work hard at school.’ These items are scored on a four-point Likert scale ranging from ‘Strongly Disagree: 1’ to ‘Strongly Agree: 4’. Items 4, 5, 15, 16, 19, 20, 22, 23, and 24 are reverse-scored. The minimum score on this scale is 30, and the maximum score is 120, where higher scores indicate higher self-efficacy. The creators reported satisfactory validity and a reliability of 0.82 using Cronbach’s alpha. Within the country, Bandak et al., [47] reported a reliability of 0.85 using Cronbach’s alpha coefficient. In the current study, Cronbach’s alpha coefficient was also used to calculate reliability, resulting in a coefficient of 0.70.

### The successful educational identity scale

Vaz and Isaacson [4] developed the Educational Identity Scale with 40 items. This study utilized 10 items related to successful educational identity, such as items 4, 5, 17, 18, 25, 28, 30, 32, 38, and 40. One of the statements included is: ‘I feel completely responsible for my learning and education.’ Higher scores indicate a more significant achievement of a successful educational identity. The scoring was conducted on a five-point Likert scale (from completely disagree: 1 to agree: 5), with possible scores ranging from a minimum of 10 to a maximum of 50. The developers reported the reliability of this tool as desirable, with a Cronbach’s alpha of 0.76. Similarly, within the country, Hajazi, et al., [48] reported a reliability of 0.76 using Cronbach’s alpha method. In this current study, Cronbach’s alpha was also utilized to calculate reliability, yielding a coefficient of 0.80.

## Findings

In this section, the assumptions of structural equation modeling were initially examined, followed by hypothesis testing. Common skewness and kurtosis statistics were calculated to assess the assumption of normality in data distribution. In this regard, Kline [49] suggests that an absolute skewness value less than three and kurtosis less than 10 indicates variables’ adherence to normality. Table 1 displays the results of the variables’ normality test.

**Table 1:** Results of variables’ normality test

Variables	Subscales	Mean	Standard Deviation	Skewness Index	Kurtosis Index
Basic	Autonomy	33.40	5.23	-0.35	0.73-
Psychological	Relatedness	35.49	4.20	-0.31	-0.57
Needs	Competence	33.53	6.12	-0.36	-0.62
Mindfulness	Observation	29.00	4.80	-0.13	-0.79
	Mindful Action	29.50	4.90	-0.14	-0.89
	Non-judgment	29.78	4.80	-0.18	-0.81
	Description	29.90	5.03	-0.24	-0.81
	Non-reactivity	27.25	5.49	0.17	-0.83
Academic self-efficacy	Texture	36.77	7.40	-0.26	0.84
	Talent	36.13	7.45	-0.13	-0.98
	Effort	11.55	2.75	-0.30	-1.07
Successful academic identity		34.78	6.31	-0.46	-0.50

Examining the results pertaining to basic psychological needs, participants indicated a moderate level of satisfaction with relatedness (M = 35.49, SD = 4.20). The distribution exhibits a slightly negatively skewed pattern (-0.31), suggesting a prevailing trend towards higher relatedness among the participants. In addition, in relation to the findings on fundamental psychological needs, it is evident that participants expressed a moderate degree of satisfaction with autonomy (M = 33.40, SD = 5.23). The distribution displays a slight negative skewness (-0.35), pointing towards a discernible inclination towards higher levels of autonomy among the participants.

Furthermore, regarding competence, participants reported a moderate level of satisfaction (M = 33.53, SD = 6.12). The distribution displays a slightly negatively skewed pattern (-0.36), while the kurtosis index (-0.62) indicates a relatively flat distribution, highlighting a nuanced perspective on competence satisfaction. Shifting focus to mindfulness, participants showed a moderate level of engagement in observation (M = 29.00, SD = 4.80), accompanied by a slightly negatively skewed distribution (-0.13), indicating a tendency towards higher levels of observation mindfulness.

Similarly, participants demonstrated a moderate engagement in mindful action (M = 29.50, SD = 4.90), with a slightly negatively skewed distribution (-0.14), emphasizing a discernible inclination towards higher mindful action. In terms of non-judgmental description, participants reported a moderate level of mindfulness (M = 29.78, SD = 4.80). The distribution is slightly negatively skewed (-0.18), signaling a trend towards higher non-judgmental description.

Moreover, participants exhibited a moderate level of engagement in non-reactivity (M = 29.90, SD = 5.03), with a slightly negatively skewed distribution (-0.24), suggesting a prevalent inclination towards higher levels of non-reactivity.

Shifting to academic self-efficacy, participants demonstrated a relatively high level of confidence in texture-related tasks (M = 36.77, SD = 7.40), along with a slightly negatively skewed distribution (-0.26), indicating a discernible inclination towards higher texture-related self-efficacy. Similarly, participants expressed a relatively high level of self-efficacy related to talent in academic tasks (M = 36.13, SD = 7.45), with a slightly negatively skewed distribution (-0.13), emphasizing a tendency towards higher talent-related self-efficacy. In the context of effort-related self-efficacy, participants reported a moderate level of engagement (M = 11.55, SD = 2.75). The distribution is slightly negatively skewed (-0.30), signifying a trend towards higher effort-related self-efficacy.

Turning to the construct of successful academic identity, participants conveyed a moderate level of perceived success (M = 34.78, SD = 6.31), with a slightly negatively skewed distribution (-0.46), indicating a discernible inclination towards a higher sense of successful academic identity.

**Fig. 2:**
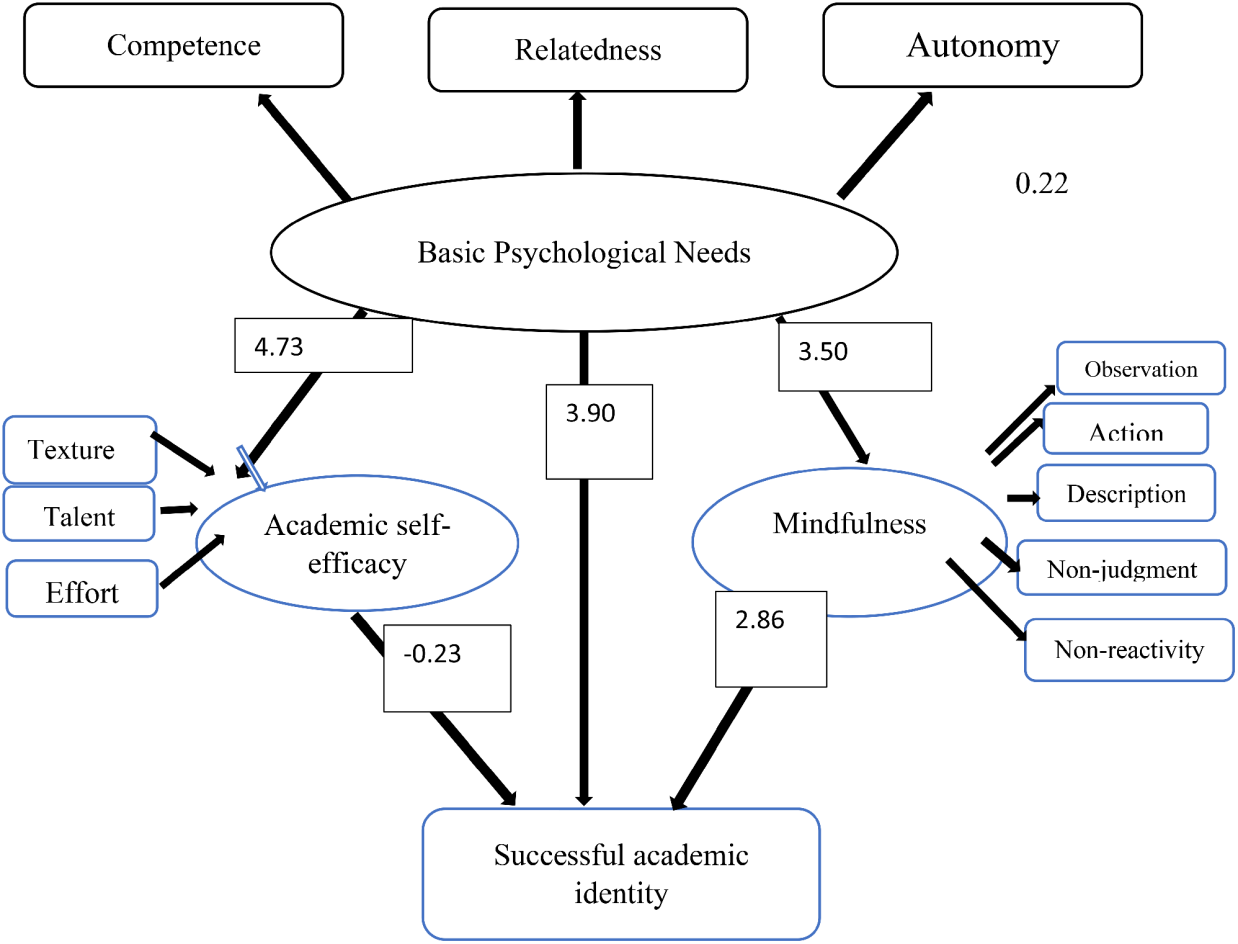
Empirical structural model (path) and measurement of successful academic identity for students based on non-standard coefficients (significant coefficients)

According to the results in the table above, all variables have adhered to the assumption of normality due to the values of kurtosis and skewness falling within a specific range. Tolerance and Variance Inflation Factor (VIF) assess multicollinearity in multiple linear regression. The tolerance statistic is a variance measure not explained by other variables, and a tolerance factor less than 0.10 indicates multicollinearity. Another issue with multicollinearity is that high correlations among predictor variables increase the standard error of their coefficients, known as variance inflation, where values above 10 indicate multicollinearity. Based on the obtained results, the tolerance factor values were: Independence = 0.71, Relationship = 0.61, Competence = 0.55, Observation = 0.61, Mindful Action = 0.59, Non-judgment = 0.71, Description = 0.50, Non-reactivity = 0.69, Texture = 0.45, Talent = 0.46, and Effort = 0.71, all of which were higher than 0.10. Additionally, the variance inflation factor values were: Independence = 1.41, Relationship = 1.64, Competence = 1.81, Observation = 1.62, Mindful Action = 1.69, Non-judgment = 1.41, Description = 1.99, Non-reactivity = 1.44, Texture = 2.22, Talent = 2.18, and Effort = 1.41, all of which were less than 10. Therefore, the assumption of no multicollinearity was met. After ensuring the necessary assumptions, structural equation modeling using the AMOS statistical software was employed for model evaluation, the results of which are observable in Fig. 2.

Various characteristics are used to evaluate the model fit. Absolutely appropriate indices are among these characteristics and serve as the primary metric for assessing the conformity of the proposed model to the collected data derived from a sample. The most important indicators within these absolute fit indices are the squared multiple correlation coefficient and the square root of the mean squared prediction error. In addition, incremental fit indices compare the estimated model to a null model, with the Tucker index being the most important indicator. Suppose that the values ​​of these comparative fit indices introduced into their decision criterion indicate an appropriate status. In this case, it validates the adaptation of the patterns and the alignment of the questions with the factors [50]. Table 2 below shows the results of the model fit indices.

**Table 2:** Model fit indices

The indices	Values of the proposed model	Values of the modified model	Acceptable range
Square root Chi (X^2^)	561.76	407.35	> 0.05
P value	0.001	0.001	
DF	242	2333	
The square root of chi over	0.060	0.047	< 0.08
Comparative fit index (CFI)	0.81	0.934	> 0.90
Root mean square error of approximation (RMSEA)	0.060	0.047	< 0.08
Tucker-Lewis Index (TLI	0.818	0.928	> 0.90
Adjusted goodness-of-fit index (PNFI)	0.678	0.671	> 0.50

**Fig. 3:**
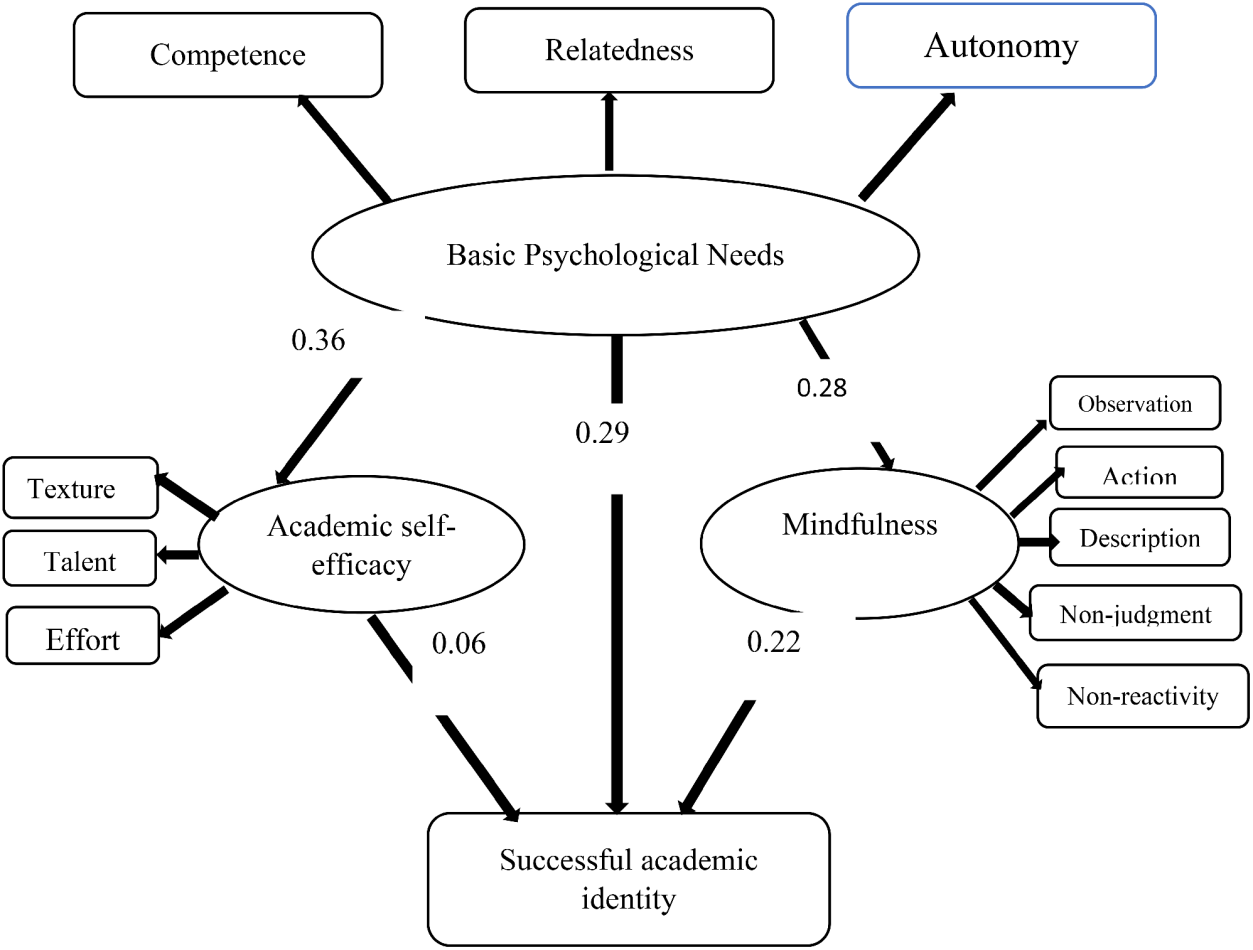
Standard coefficients of paths in the modified model

A root mean square error of approximation (RMSEA) index of less than 0.08 indicates a better model fit. CFI and TLI indices evaluate model improvement by comparing a model to an independent model where there are no relationships between variables as suggested by the researcher. TLI demonstrates the level of model fit. Both indices range between zero and one; The closer their values ​​are to one, the better the model fits. The values ​​of these two indices in the proposed model were below 0.9, so the measurement models had to be adjusted accordingly. In order to improve the values ​​of these two indices, model modification statements suggested by the software were used. This included establishing relationships between the existing errors in each variable and modifying the proposed model as mentioned, as shown in Fig. 3; Table 3.

**Table 3:** Measurement parameters of direct and indirect relationships of variables

Independent variable	Mediating variable	Dependent variable	Β	T	P	Results
Basic psychological needs	--------	Successful academic identity	0.29	3.90	0.001	Confirmed
Basic psychological needs	--------	Mindfulness	0.28	3.50	0.001	Confirmed
Basic psychological needs	--------	Academic self-efficacy	0.36	4.73	0.001	Confirmed
Mindfulness	--------	Successful academic identity	0.22	2.86	0.001	Confirmed
Academic self-efficacy	--------	Successful academic identity	-0.02	-0.235	-0.814	Rejected
Basic psychological needs	Mind-fulness	Successful academic identity	0.26	2.20	0.001	Confirmed
Basic psychological needs	Self-academic efficacy	Academic successful identity	0.26	-0.234	0.16	Rejected

Firstly, the study addressed the relationship between Basic Psychological Needs and students’ mindfulness, confirming Hypothesis 1 (β = 0.28, T = 3.50, *P* = 0.001). Secondly, Hypothesis 2 was supported, revealing a significant positive impact of Basic Psychological Needs on students’ academic self-efficacy (β = 0.36, T = 4.73, *P* = 0.001). Thirdly, the study affirmed Hypothesis 3, demonstrating that Basic Psychological Needs have a significant effect on students’ successful academic identity (β = 0.29, T = 3.90, *P* = 0.001). Moreover, the research delved into the mediating role of mindfulness. Hypothesis 4 was confirmed, indicating that Basic Psychological Needs influence students’ successful academic identity through the mediating variable of mindfulness (β = 0.26, T = 2.20, *P* = 0.001). Conversely, Hypothesis 5, proposing a mediating role for academic self-efficacy, was not supported (β = -0.02, T = -0.235, *P* = 0.814). Furthermore, the study explored the direct impact of mindfulness on students’ successful academic identity, confirming Hypothesis 6 (β = 0.22, T = 2.86, *P* = 0.004). Lastly, Hypothesis 7, suggesting a direct effect of academic self-efficacy on successful academic identity, was rejected (β = 0.28, T = -0.234, *P* = 0.001). In summary, the findings underscore the significance of Basic Psychological Needs, confirm the mediating role of mindfulness, and emphasize the direct influence of mindfulness on students’ successful academic identity. However, the study did not find support for the proposed mediating role of academic self-efficacy in this context.

.

## Discussion

This research presented a structural model of successful academic identity based on fundamental psychological needs mediated by mindfulness and academic self-efficacy. The results have shown that fulfilling fundamental psychological needs leads to an increase in successful academic identity. In other words, the more the needs for autonomy, relatedness, and competence are satisfied, the easier it is to achieve a successful academic identity. Mindfulness is a variable that influences the relationship between fundamental psychological needs and successful academic identity, enhancing the impact of basic psychological needs on successful academic identity. In other words, mindfulness amplifies the effect of fundamental psychological needs on successful academic identity. However, as another mediator in the model, academic self-efficacy played a minor role and could not meaningfully impact the relationship between fundamental psychological needs and successful academic identity. In other words, academic self-efficacy did not significantly influence the increase or decrease in the impact of basic psychological needs on successful academic identity.

The first result of the research demonstrated a positive impact of fundamental psychological needs on successful academic identity, leading to an increase in successful academic identity. This aligns with the findings of prior studies [16–19]. According to the Self-Determination Theory, when they meet the three primary needs of autonomy, relatedness, and competence to a satisfactory level, adolescents develop a high readiness to attain a successful academic identity. Achieving autonomy, they are not driven by the pursuit of others’ values and goals, nor are they subjected to pressures to conform to externally set educational values. Instead, they make their own choices and decisions, ultimately shaping a successful academic identity [51]. This text discusses the importance of interaction and communication in identifying goals and values, particularly in academic areas. Individuals select their objectives by observing the performance of others through communication and interaction. Successful individuals use these methods; in other words, by engaging with others, they celebrate both academic failures and successes. Consequently, by comparing these two groups and evaluating the reasons behind their failure and success, individuals gain better self-awareness and choose better paths [52].

Another finding indicated that satisfying basic psychological needs positively and significantly impacts mindfulness. In other words, meeting the needs for autonomy, relatedness, and competence significantly influences mindfulness. Conversely, if the satisfaction of these needs for autonomy, relatedness, and competence is low in an individual, their mindfulness will also be diminished. This finding is consistent with the results of Wilson et al [27]. In elucidating this finding, it can be said that basic psychological needs, as implied by their name, represent the most basic requirements of an individual. By fulfilling these needs, an individual meets their mental needs. Consequently, when in favorable psychological conditions [53], the individual addresses challenges in any domain fully committed. According to the Self-Determination Theory, such individuals focus on problem-solving by mastering their emotions, thoughts, and behaviors and strive for optimal performance. Therefore, when psychological needs are satisfied, performance improves, and intrusive psychological variables that can detract from mental focus are absent. Another study result showed that the satisfaction of basic psychological needs leads to increased academic self-efficacy. In other words, when an individual’s needs for autonomy, relatedness, and competence are satisfied, their academic self-efficacy also increases. This finding aligns with the results of Niemie and Ryan [30]. Elaborating on this finding based on the Self-Determination Theory, it can be said that an individual demonstrating a sense of autonomy displays higher agency and strives independently and directly to exert influence on impactful phenomena and issues, leading to an increase in self-efficacy. By establishing connections, they compare others’ perspectives, abilities, and performances. They learn from these comparisons of successful and unsuccessful performances, viewing the outcomes of similar individuals and feeling that they, too, can achieve similar performances, thereby enhancing their self-efficacy [54]. Furthermore, experiencing competence in the learner leads to the belief in their ability to positively impact the environment, fostering a high sense of self-efficacy [55]. Another subsequent result of the research demonstrated that mindfulness contributes to an increase in academic identity, indicating that heightened mindfulness effectively contributes to achieving a successful academic identity. This result aligns with the findings of Arici Özcan and Vural [26] and Yu and Li [28]. In explaining this finding, it can be said that mindfulness, through focused attention and conscious and deliberate action, enhances the learner’s cognitive capacity by overcoming cognitive barriers involved in problem-solving [26]. Witnessing improved performance strengthens the learner’s capabilities, motivating them to apply similar strategies in similar situations consistently. Over time, a mental framework is constructed, leading to increased influence and competence in task execution, significantly impacting achieving a successful academic identity. Another research finding indicated that academic self-efficacy does not significantly increase or decrease successful academic identity. In other words, it was revealed that academic self-efficacy does not influence attaining a successful academic identity. This finding contrasts with studies by Caraway, et al. [32] and Sansone and Harackiewicz [31]. This result can be elucidated by structural equation modeling, which determines causal relationships. However, the studies mentioned utilized correlation, making it impossible to infer causal relationships; in other words, it can only be said that variables are related. Additionally, the emphasis on the mediational role of self-efficacy in most variables [56–59] was a significant reason for the application of this variable as a mediator. Based on the findings, it can be argued that contrary to the prevalent view among researchers [57–59] self-efficacy cannot always serve as a mediator variable in research. Instead, it might be influenced by various factors, including academic identity. Therefore, it is likely that successful academic identity leads to improved performance by utilizing bo. In the findings related to the mediational roles in the model, it became apparent that despite mindfulness, basic psychological needs will have a greater impact on successful academic identity. To expound on this result, alongside fulfilling basic needs, other factors like mindfulness can contribute to achieving a successful academic identity.

The satisfaction of psychological needs leads to autonomy, establishment of connections, drawing from others’ experiences, and an enhanced understanding of competencies. These factors directly facilitate the attainment of a successful academic identity. However, mindfulness ensures that, beyond the mental readiness resulting from meeting needs, the learner remains conscious of their thoughts and actions moment by moment. With a cohesive cognitive organization, they strive to employ strategies that will yield the best results when faced with a task or challenge. the exploratory behavior and commitment, thereby enhancing academic self-efficacy. The study’s outcome indicated that the presence or absence of academic self-efficacy does not play a role in the impact of basic psychological needs on successful academic identity. It is evident that when a mediating variable lacks a relationship with the inner or outer variables or is disconnected from any of them, it cannot serve as a mediator. Therefore, contrary to the research assumption and scholars’ beliefs, self-efficacy does not consistently act as a mediator; rather, it might be an internal variable. Based on the research findings, it is essential for families and schools, as two basic and influential institutions in the socio-psychological growth of learners, to pay attention to their basic psychological needs and create conditions that enhance independence, increase interactions with learners, and instill a sense of competence in them. Therefore, family education sessions held within schools are suggested to address this matter and elucidate the underlying factors contributing to successful academic identity. Additionally, senior educational managers should take measures to allocate time for teachers and educators to teach students not just academic subjects but also how to learn, act, make choices, and overall, how to grow alongside their educational activities. Researchers interested in this field are recommended to utilize successful academic identity as a mediating variable and academic self-efficacy as an intrinsic variable in future research to determine their mutual impact and influence on each other.

## Concluding remarks

In summation, this research is a testament to the intricate interplay of factors that coalesce to shape successful academic identity. The findings resoundingly affirm the indispensability of fulfilling fundamental psychological needs, especially autonomy, relatedness, and competence, in creating an environment conducive to the development of a successful academic identity. Mindfulness emerges as a critical amplifier, enhancing the impact of basic psychological needs on academic identity, while academic self-efficacy, albeit playing a mediating role in the model, surfaces with a limited impact within this context.

The study posits profound implications for educational settings, advocating for the creation of conditions that foster independence, elevate interactions with learners, and instill a profound sense of competence. It is recommended that family education sessions comprehensively address these factors, shedding light on the nuanced elements that contribute to the development of a successful academic identity. Moreover, educational institutions should proactively allocate time for educators to impart not only academic knowledge but also essential life skills, equipping students to navigate their educational journey with resilience and self-awareness. Future research endeavors in this domain are encouraged to delve into the reciprocal impact of successful academic identity as a mediating variable and academic self-efficacy as an intrinsic variable. While acknowledging the limitations inherent in questionnaire-based data collection, this research provides a robust platform for further exploration into the intricate dynamics of psychological factors influencing students’ academic experiences.

## Data Availability

The data will be made available upon the request from corresponding author.

## References

[1] Berk L. Child Development Psychology. Vol 2. Translated by Yahya Seyed Mohammadi; Arasbaran Publications; 2007.

[2] Erikson EH. Identity: Youth and Crisis. Norton; 1968.

[3] Mohammadi Azad F. Prediction of Academic Procrastination Based on Academic Resilience and Academic Identity of Female Students at Qom University in the Academic Year 2018–2019. Master’s Thesis, Qom University; 2019.

[4] Was CA, Isaacson RM (2008). The development of a measure of academic identity status. J Res Educ.

[5] Sekiwu D, Ssempala F, Naluwemba F. Investigating the relationship between school attendance and academic performance in universal primary education. The case of Uganda; 2020.

[6] Aldahlawi MMY. (2020). *Examining the Relationship of Student Characteristics on Engagement to Predict Academic Gains of Saudi Female International Students in the United States of America* (Doctoral dissertation, Morgan State University).

[7] Marcia JE (1966). Development and validation of Ego-identity status. J Pers Soc Psychol.

[8] Glasser W. Reality therapy: a New Approach to Psychiatry. Harper & Row; 1965.

[9] Shafieeabadi A, Abdollah, Naseri G. Counseling and psychotherapy theories. University Press Center; 2013.

[10] Deci EL, Ryan RM, Self-Determination Theory (2008). A macro theory of human motivation, Development, and Health. J Can Psychol.

[11] Ryan RM, Deci EL. Self-determination theory: Basic Psychological needs in motivation, Development, and Wellness. The Guilford; 2017.

[12] León J, Núñez JL (2013). Causal ordering of Basic Psychological needs and well-being. Soc Indic Res.

[13] Evelein F, Korthagen F, Brekelmans M. Fulfillment of The Basic Psychological Needs of Student Teachers During Their First Teaching. 2008.

[14] Gazla S. Psychological Health: Exploring the Relationships between Psychological Flexibility, Basic Psychological Needs Satisfaction, Goal Pursuits, and Resilience. Doctoral Dissertation, the West of England University; 2015.

[15] Arnone MP, Reynolds R, Marshall T (2009). The effect of early adolescents’ psychological needs satisfaction upon their perceived competence in information skills and intrinsic motivation for Research. School Libr Worldw.

[16] Faye C, Sharpe D (2008). Academic motivation in University: the role of Basic Psychological needs and identity formation. Can J Behav Sci.

[17] Luyckx K, Vansteenkiste M, Goossens L, Duriez B (2009). Basic need satisfaction and identity formation: bridging self-determination theory and process-oriented identity research. J Couns Psychol.

[18] Rogers M, Tannock R (2018). Are classrooms meeting the basic psychological needs of children with ADHD symptoms? A self-determination theory perspective. J Atten Disord.

[19] Moghaddas Z, Tabaei Z (2020). The role of Basic Psychological needs in ethical identity. J Ethics Sci Technol.

[20] Baer RA (2003). Mindfulness training as a clinical intervention: a conceptual and empirical review. Clin Psychol Sci Pract.

[21] Kabat-Zinn J (2003). Mindfulness-based interventions in Context: past, Present, and Future. Clin Psychol Sci Pract.

[22] Kabat-Zinn J. Mindfulness Meditation for Everyday Life. Hyperion; 1994.

[23] Lengacher CA, Kip KE, Barta M, Post-White J, Jacobsen PB, Groer M, Shelton MM (2012). A pilot study evaluating the effect of mindfulness-based stress reduction on psychological status, physical status, salivary cortisol, and interleukin-6 among advanced-stage cancer patients and their caregivers. J Holist Nurs.

[24] Shapiro SL (2009). The integration of mindfulness and psychology. J Clin Psychol.

[25] Baumgartner PD, Schneider, PhD TR (2023). A randomized controlled trial of mindfulness-based stress reduction on academic resilience and performance in college students. J Am Coll Health.

[26] Arici Özcan N, Vural Ö (2020). The Mediator Role of thriving in the relationship between self-efficacy and mindfulness in Middle-Adolescence Sample. Educational Sciences: Theory Pract.

[27] Wilson AJ, Liu Y, Keith SE (2012). Transformational teaching and child psychological needs satisfaction, motivation, and engagement in elementary school physical education. Sport Exerc Perform Psychol.

[28] Yu C, Li X, Zhang W (2015). Predicting adolescent problematic online game use from teacher autonomy support, basic psychological needs satisfaction, and school engagement: a 2-year longitudinal study. Cyberpsychology Behav Social Netw.

[29] Ryan RM, Deci EL (2000). Self-determination theory and the facilitation of intrinsic motivation, social development, and well-being. Am Psychol.

[30] Niemiec CP, Ryan RM (2009). Autonomy, competence, and relatedness in the classroom: applying self-determination theory to educational practice. Theory Res Educ.

[31] Sansone C, Harackiewicz JM (2000). Intrinsic and extrinsic motivation: the search for optimal motivation and performance.

[32] Caraway K, Tucker CM, Reinke WM, Hall C (2003). Self-efficacy, goal orientation, and fear of failure as predictors of school engagement in high school students. Psychol Sch.

[33] Bassi M, Steca P, Fave AD, Caprara GV (2007). Academic self-efficacy beliefs and quality of experience in learning. J Youth Adolesc.

[34] Bandura A, Freeman WH, Lightsey R (1999). Self-efficacy: the exercise of control. J Cogn Psychother.

[35] Pajares F. Self-efficacy beliefs in academic settings. Review of Educational Research. 1996;66:543–578. Caraway, Sansone and Harackiewicz.

[36] Wright SL, Jenkins-Guarnieri MA, Murdock JL (2012). Career development among first-year college students: College self-efficacy, student persistence, and academic success. J Career Dev.

[37] Galyon CE, Blondin CA, Yaw JS, Nalls ML, Williams RL (2012). The relationship of academic self-efficacy to class participation and exam performance. Soc Psychol Educ.

[38] Arianpour H, Hajazi E, Azhaei J, Ghalamali Lavasani M (2019). Academic identity of Iranian students: a qualitative study. Psychol Mag.

[39] Dunham N. The academic identity of students in the early childhood field-based on initial teacher education. Unpublished thesis submitted in fulfillment of the requirements for the degree of Doctor of Philosophy (Education), Unitec Institute of Technology. Experiences. Teach Teach Educ. 2016;24:1137–1148.

[40] Tabachnick BG, Fidell LS (2001). Using Multivariate statistics.

[41] La Guardia JG, Ryan RM, Couchman CE, Deci EL (2000). Within-person variation in security of attachment: a self-determination theory perspective on attachment, need fulfillment, and well-being. J Pers Psychol.

[42] Besharat MA, Ranjbar Kalagari E (2013). Psychometric scale of satisfaction of Basic Psychological needs: reliability, validity, and factor analysis. J Educ Meas.

[43] Gholamali Lavasani M, Mirhosseini FA, Mohtarinassab Z, Ramesh S (2019). The Mediating Role of Self-Integration in the relationship between Basic Psychological needs and cognitive emotional regulation strategies in adolescents. Psychol Dev.

[44] Baer RA, Smith GT, Hopkins J, Krietemeyer J, Toney L (2006). Using self-report assessment methods to explore facets of mindfulness. Assessment.

[45] Ahmadvand Z, Heidari Nasab L, Shaeiri MR (2012). Explaining Psychological Well-being based on Mindfulness Components. Health Psychol.

[46] Jinks J, Morgan V (1999). Children’s Perceived Academic Self-Efficacy: an Inventory Scale. Clear House.

[47] Bandak M, Maleki H, Abbaspour A, Ebrahimi Ghavam S (2015). The Effect of Life Skills Education on students’ academic self-efficacy. J Educ Psychol.

[48] Hajazi E, Amani H, Yazdani MJ (2011). Assessment of Psychometric properties of the academic identity Status Questionnaire in Iranian students. Psychol Methods Models.

[49] Kline RB (2011). Principles & practice of structural equation modeling.

[50] Abarshei A, Hosseini SY (2012). Structural equation modeling.

[51] Deci EL, Ryan RM (2002). Handbook of self-determination research.

[52] Zhen R, Liu RD, Ding Y, Wang J, Liu Y, Xu L (2017). The mediating roles of academic self-efficacy and emotions in the Relationship between Basic Psychological needs satisfaction and learning Engagement among Chinese adolescent students. Learn Individ Differ.

[53] Deci EL, Ryan RM (2000). The what and why of goal pursuits: human needs and the self-determination of Behavior. Psychol Inq.

[54] Akomolafe M, Ogunmakin A, Fasooto G. The role of academic self-efficacy, academic motivation and academic self-concept in predicting secondary school students’ academic performance. Journal of Educational and Social Research; 2013.

[55] Zhen R, Liu RD, Ding Y, Wang J, Liu Y, Xu L (2017). The mediating roles of academic self-efficacy and academic emotions in the relation between basic psychological needs satisfaction and learning engagement among Chinese adolescent students. Learn Individual Differences.

[56] Macakova V, Wood C (2022). The relationship between academic achievement, self-efficacy, implicit theories and basic psychological needs satisfaction among university students. Stud High Educ.

[57] Pajares F (2002). Self-efficacy beliefs in academic contexts. Rev Educ Res.

[58] Valentine JC, DuBois DL, Cooper H (2004). The relation between self-beliefs and academic achievement: a systematic review. Educ Psychol.

[59] Pajares F, Schunk DH, Marsh HW, Craven RG, McInerney DM (2005). Self-Efficacy and Self-Concept beliefs: jointly contributing to the quality of Human Life. International advances in self-research.

