# Correction: Structural modeling of Chinese students’ academic achievement identity and basic psychological needs: do academic self-efficacy, and mindfulness play a mediating role?

**DOI:** 10.1186/s40359-024-01866-8

**Published:** 2024-07-03

**Authors:** Shuya Chen

**Affiliations:** Basic Courses Teaching Department, Henan Judicial Police Vocational College, 450016 Zhengzhou, Henan China

**Correction: BMC Psychology (2024) 12:142**

10.1186/s40359-024-01571-6

Following publication of the erratum, the authors flagged that there were a number of errors in the article text that had occurred when translating an earlier, Chinese version of the article into English. Specifically, the authors flagged that the following corrections needed to be made to their published article:

In the “background” of the abstract, please replace “first-grade high school” with “College”;

In the “Method” section of the abstract, change “undergraduate” to “College”;

In the “Findings” section of the abstract, replace “high school” with “college”;

In the “Conclusions” section of the abstract, change “undergraduate” to “college”;

In the “Introduction” section, change “high school” to “college” and, if possible, remove the following sentence: “Moreover, the looming challenge of college entrance exams, identified as pivotal educational hurdles, underscores the difficulty in determining the factors contributing to academic identity formation”;

In the “Method” section of the main text, replace “high school” with “college”

The published article has since been updated accordingly. The authors thank you for reading this erratum and apologize for any inconvenience caused.

